# Task sharing for the management of non-communicable diseases in a humanitarian setting: An innovative approach in a low-middle income country (LMIC)

**DOI:** 10.1016/j.puhip.2025.100643

**Published:** 2025-07-25

**Authors:** Myrna Doumit, Sahar Masri, Iza Ciglenecki, Manuel Albela

**Affiliations:** a American University of Beirut, Hariri School of Nursing, Beirut, Lebanon; b Médecins sans Frontières Operational Center of Geneva (OCG), Beirut, Lebanon; c Médecins sans Frontières Operational Center of Geneva (OCG), Lebanon

## Abstract

The World Health Organization (WHO) defines task shifting as the balanced reallocation of tasks from highly experienced professionals to those with more limited training, thus allowing the existing human resources to be used more efficiently. In Lebanon, there is no documented evidence yet of task sharing between physicians and nurses in the usual primary healthcare setting, let alone for Non-Communicable Disease (NCD) management.

**Objective:**

This study aims to explore the experiences of healthcare providers and patients regarding task sharing.

**Study design:**

This study employed a qualitative descriptive design, utilizing a cross-sectional approach.

**Method:**

Qualitative data collection started in April–May 2022. Data collection was conducted with three focus groups: nurses (n = 9), patients (n = 11), and physicians (n = 5) from two clinics in two different rural areas, using purposeful sampling. A thematic analysis method, as described by Braun and Clarke, was used to guide the analysis.

**Results:**

This study yielded four themes: An innovative approach to care, A prevailing culture of trust and collaboration, a Synergistic Outcome, and A Call for Improvement.

**Conclusion:**

This study has identified an innovative approach to care, as demonstrated by the practices performed at the two clinics. Task shifting is a means of ensuring nurses' satisfaction. Therefore, it may positively impact retention at a time when we are witnessing an unprecedented migration of nurses from low- and middle-income countries (LMICs) to high-income countries (HICs). Interprofessional education needs to be reinforced at the undergraduate level to enhance collaboration among health care workers after graduation. At the policy level, considerable work is necessary to ensure that all stakeholders’ voices are represented at the decision-making table and heard.

## 1 Introduction

The World Health Organization (WHO) defines task shifting as the reallocation of functions from highly experienced professionals to those with less training, thereby using human resources more efficiently [1]. WHO categorizes task shifting into four types based on which cadres assume new responsibilities. For example, task shifting type II involves nurses taking over tasks initially performed by doctors [1]. Task-sharing models are tailored to a country's context, depending on the available mix of skilled health workers, disease burden, and healthcare financing schemes [1,2]. In low-income countries such as Malawi, Tanzania, and Ethiopia, task shifting and task sharing have improved access to emergency obstetric and postnatal care by non-physician health workers [3].

In Lebanon, there is no documented evidence of task sharing between physicians and nurses in primary healthcare, particularly for the management of Non-Communicable Diseases (NCDs). In this study, task sharing at two clinics in a rural area of Lebanon involves a baseline consultation by a doctor, followed by referrals to an NCD nurse for patient education and support. Stable patients may have follow-up consultations with nurses, reducing the frequency of doctor consultations. This innovative approach is unique in Lebanon's healthcare system.

A unique study on nurse-physician collaboration in Lebanese hospitals revealed that physicians often do not favor collaborative relationships and view nurses primarily as implementers of physicians' orders [4]. This lack of professional cooperation, poor compensation, and limited professional development negatively impact job satisfaction and nurse retention. Lebanon's fragmented health system faces imbalances between rural and urban areas in health personnel, exacerbated by the influx of Syrian refugees seeking primary care. Despite international support, the health system remains fragmented with parallel services. Exploring task sharing in Lebanon's primary healthcare has not been thoroughly discussed in the literature, despite the significant challenges in human resources and the need for sustainable public health strategies amid ongoing crises.

The study aims to explore the experiences of healthcare providers and patients with task sharing, emphasizing its potential as a complementary model of primary healthcare in resource-limited areas. This study took place in Irsal, a town in northeastern Lebanon near the Syrian border. This town has become a significant hub for Syrian refugees since the outbreak of the Syrian Civil War in 2011. The city has faced various challenges as it hosts a large number of displaced Syrians, with the dynamics of the refugee presence impacting the local population and the broader region. In Irsal, healthcare facilities for Syrian refugees and Lebanese living in the region face significant challenges due to the high demand for services, limited resources, and the strain on the local healthcare system.

This study aimed to explore the experiences of healthcare providers and patients regarding task sharing. The importance of this study lies in its consideration of a complementary model for primary healthcare provision, one that could be implemented in areas where the health workforce and other resources are limited.

## 2 Methods

### 2.1 Design

The study employed a qualitative descriptive design with a cross-sectional approach to subjectively describe participants' experiences. This research method aligns with constructionism and critical theories, viewing reality as dynamic and context-dependent [5].

### 2.2 Data collection

Qualitative data were collected from three focus groups, comprising nurses, patients, and physicians, at two rural clinics through purposeful sampling from April to May 2022 (Table 1). After receiving ethics approval, clinic managers explained the study to healthcare workers, who volunteered to participate. Social workers approached patients, and upon agreeing, they were contacted by the principal investigator (PI) for written consent.

**Table 1:** Characteristics of participants in FGDs.

	Patients' FGD	Nurses' FGD	Physicians' FGD
Males (n)	6	2	4
Females (n)	5	7	1
Mean Age (±s.d)	Not available	35.4 (±7.3)	40.8 (±11.2)
Mean years of experience with MSF (±s.d)	Not available	6.4 (±2.9)	6.2 (±3.2)

Each focus group consisted of 5–8 participants with diverse socio-cultural backgrounds, gender, educational levels, and years of experience. Separate focus groups were conducted for each clinic's nurses, physicians, and patients. Each focus group session started by collecting demographic information, followed by a grand tour question related to the process at the clinic, followed by probing for every idea, such as “Tell me more about it, give me more examples, can you elaborate more on it”. Participants were asked to answer and provide examples. Saturation within focus groups for each question was sought to ensure thorough coverage. Saturation was reached when no new ideas would surface. Furthermore, recognizing that participants in focus groups may choose to provide socially pleasing answers, the investigator clarified at the beginning of each session that she was concerned about individuals' actual thoughts, feelings, and experiences. Furthermore, equivalence, internal consistency, and validity of the process were maintained by one investigator, who led the discussion with one group and across groups. Sessions lasted about 50–60 min. Data saturation was reached when no new ideas emerged. An expert transcribed the data verbatim, ensuring conceptual equivalence through translation and back-translation.

### 2.3 Data analysis

Thematic analysis, following Braun and Clarke's method [6], guided the study. The process involved five steps: familiarization with the data, generating initial codes, sorting the codes, defining and naming the codes, and reporting the data. NVivo version 13 was used to help organize and analyze the data.

### 2.4 Rigor

The study ensured rigor through credibility, dependability, confirmability, and transferability, using peer debriefing, triangulation, and a clear audit trail [7,8].

### 2.5 Confidentiality and consent

Participants' confidentiality was maintained through the use of coded transcripts and the secure storage of consent forms. Participation was voluntary, with the option to withdraw at any time without affecting one's job or access to care.

### 2.6 Ethical approvals

The study received ethical approval from the MSF Ethics Review Board and the Institutional Review Board (IRB) of the principal investigator's workplace.

## 3 Results

The study identified four themes, with all participants supporting the task-sharing approach. Each group of participants valued this novel approach from a different angle. It is worth noting that patients were from Syrian and Lebanese nationalities. Since 2011, more than a million refugees from Syria have arrived in Lebanon. With a population of just over four million, today, Lebanon has the world's highest number of refugees per capita. Most refugees remained in camps in rural areas of Lebanon, where they benefited from the care provided in local clinics.

### 3.1 Theme 1: an innovative approach to care

Participants described the process at both clinics as innovative, highlighting the enhanced role of nurses in patient care, which improved care quality and patient satisfaction.

Nurse 1, Clinic 1**: *… the relationship between physicians and us in this clinic is different than any other place. It is built on trust and respect. And this is reflected in the care provided to patients…***

Patient 1, Clinic 2: ***The services offered here are distinct from those found at any other facility. It's even different than Syria. The nurses here ask you detailed questions, and they care about you. We first come to see the nurse, and sometimes we do not know the physician. But we are satisfied and happy because we are receiving good care…***

Physician 1, clinic 1***: … We have a standard protocol for stable patients. They come for two visits to see the nurses and one visit to see the physician. We discuss all cases with nurses. This collaborative relationship with nurses is highly beneficial….***

### 3.2 Theme 2: A culture of trust and collaboration

The collaborative relationship between nurses and physicians fostered trust among all parties, enhancing the quality of care and patients’ sense of being treated with dignity and respect.

Patient 4, Clinic 2:” … ***Every month, they assess you from head to toe. Nurses and physicians ask you detailed questions. You feel that they genuinely care about you, as if you were a family member. This is a good feeling, and it's different from any other clinic I've visited here and in Syria. I visited other clinics in the area, and they are entirely different. They do not give you the focused care that you receive here. And this is what makes them unique*** …”

Nurse 3, clinic 1: **“… our role with patients increased a lot, and patients have more trust in us …”**

Physician 2, clinic 1: “***… the collaboration between physicians and nurses decreases the waiting time for patients and gives us more time to see the complicated cases … we do a monthly educational meeting with the nurses….”***

### 3.3 Theme 3: Synergistic Outcome

Collaboration and trust allowed effective use of time and resources, benefiting both healthcare providers and patients. Patients appreciated the organized and thorough care process, while physicians valued the support of nurses.

Patient 6, clinic 1: “ ***…. I have lived in Lebanon for 10 years and have been visiting this place for 9 years. I visited many clinics, but this one is the best. The care is well-coordinated, and they have a humane approach. They care about you as a person; they give us special attention. They do not just give us our medication, no; they assess us before, then they provide the meds…”***

Physician 3, Clinic 1***: “… the collaborative relationship with the nurses is beneficial for us and them. I can describe it as a complementary relationship that benefits patients. I am confident that all patients are delighted with the care they receive, and I am equally satisfied as a physician because I can rely on the nurses who are doing a good job. This gives me more time to concentrate on the complicated cases or to see more patients per day….”***

Nurse 5, clinic 1***: “… the professional relationship that we have with the physicians in this clinic and the level of collaboration boosted our morale and made patients have trust in us, which is very rewarding. When patients see how physicians confirm our teaching or instruction, they develop trust in us. It is something that we do not see in other places. Gaining patient trust in us (nurses) is something essential…”***

### 3.4 Theme 4: A Call for Improvement

Despite being satisfied with the quality of care, participants suggested improvements, including home visits for patients who are unable to afford transportation, increased physical space for privacy, continuous training for healthcare workers, and improved infrastructure and medication availability.

Patient 7, clinic 2: “***…I wish they could send someone to assess me at home. I do not have the money to come every month. This time, I took money from friends to pay for the transportation. I wish they could come to people who cannot come…”***

Nurse 6, clinic 2: ***“…I wish we could have more time to spend with patients, but unfortunately, we have a big daily load. Additionally, I would like the physical space to be wider to accommodate more patients and provide more privacy during consultations. Also, we desire to have more workshops and continuous training to keep on improving our knowledge…”.***

Physician 4, clinic 1***:” … I wish we could introduce the electronic file. This would facilitate the job between us and the nurses. Additionally, I would like to see a dietitian on the team. We need to have a dietitian in the clinic. Additionally, the infrastructure needs improvement. There is no confidentiality while talking with patients. Rooms are divided by Gibson boards, which is not right …*** “

## 4 Discussion

The results of this innovative study within MSF clinics in Lebanon highlighted the positive impact of task shifting on Health Care Workers (HCW) satisfaction, knowledge, and performance, and patient satisfaction in primary healthcare settings. All stakeholders viewed the new approach as innovative. Patients who were initially unsure of the role of nurses in primary health care (PHC) appreciated the outcome of this approach, which resulted in improved quality of care for them. However, this limited knowledge related to nurses' role in PHC that needs improvement is comparable to the results of other studies, which also reported patients’ lack of knowledge about nurses' fundamental role [[9], [10], [11], [12], [13], [14], [15]]. Both physicians and nurses viewed physician-nurse substitution and collaborative practice as a means of improving the care process and developing a professional relationship founded on trust and respect. Similar results were highlighted by other studies as well [[15], [16], [17], [18], [19], [20]].

A close physician-nurse relationship, characterized by trust and mutual respect, supports nurses in enhancing and expanding their roles, leading to increased satisfaction and self-esteem, crucial factors in retaining qualified nurses. The physician-nurse relationship was viewed as an enabler of role development and collaborative work among nurses, resulting in an improved work environment and enhanced patient care services. Professional trust, mutual respect, and a close working relationship with physicians permitted nurses to advance their role. This was linked to feeling 'valued,' 'trusted,' and 'appreciated.' This trusting relationship between physicians and nurses also reflected positively on the nurse-patient relationship, particularly in a country where nurses are not generally viewed by patients as trustworthy as physicians. In return, patients showed increased trust in nurses, which had a positive impact on the nurses. On the other hand, nurses at the centers who worked in different locations and did not have a close relationship with their physicians' colleagues spoke of feeling 'totally unsupported' and 'powerless', which affected their self-esteem and job satisfaction. Physicians valued the role nurses played and felt that it gave them more time to concentrate on complicated cases and see more patients. Studies by Dierick-van Daele [21] and Hamel [22] reported that physicians considered task sharing as a means of relieving their workload. In settings where physicians' revenues came from fee-for-service payments, physician-nurse substitution was perceived as a problem and a threat to physicians’ financial status (31–32). This trustful relationship between physicians and nurses created a synergistic positive impact on the work environment that all stakeholders felt and benefited from.

Despite the satisfaction expressed by the stakeholders of this study, they also indicated a need for improvement when given the opportunity. The requested change affected multiple aspects, primarily the training component. Nurses requested more workshops to expand their knowledge base, and physicians appreciated the common training sessions with nurses. It is worth noting that the Lebanese population has grown by up to 25 % since the beginning of the Syrian crisis in March 2011, and this sharp increase in levels has put pressure on Lebanese facilities, facilities already weakened by a long-lasting internal crisis, and exacerbated by current political unrest and economic uncertainty [23]. The primary healthcare system in Lebanon struggled to meet the challenges imposed by the increased number of refugees who fled into the country; however, without the necessary support and economic stability, it failed to expand and meet the needs of patients and healthcare workers. This additional pressure was translated into HCW burnout and patients' (both host and refugees) complaints of poor quality of care and unsatisfied health care needs [23].

## 5 Conclusion

This study has identified an innovative approach to care, as demonstrated by the practices performed at the two clinics. This new approach within the prevailing healthcare system could be an alternative to the retention strategy for physicians and nurses in LMIC, as it provides opportunities for growth and development. Several recommendations can be made at the practice, education, and policy levels to help meet the challenges faced by the healthcare system in Lebanon. Task shifting is a means of ensuring nurses' satisfaction. Therefore, it may positively impact retention at a time when we are witnessing an unprecedented migration of nurses from low- and middle-income countries (LMICs) to high-income countries (HICs). Interprofessional education needs to be reinforced at the undergraduate level to enhance collaboration among healthcare workers (HCWs) after graduation. At the policy level, considerable work is required to ensure that all stakeholders’ voices are represented at the decision-making table and heard. This study also lays the groundwork for future studies, both locally and regionally. Though some of the findings and recommendations may be situation-specific, others undoubtedly would apply to other countries in the region.

## Ethical approval

Ethical approval was obtained from the relevant institutional review board. IRB #: LAU.SON.MD2.10/Nov/2021.

## Funding

This study was funded by Médecins sans frontiers. The funding agency did not interfere in any aspect of the research.

## Conflict of interest

All authors declare no conflict of interest.
